# Patient Acceptance of Prescribed and Fully Reimbursed mHealth Apps in Germany: An UTAUT2-based Online Survey Study

**DOI:** 10.1007/s10916-023-01910-x

**Published:** 2023-01-27

**Authors:** Marie Uncovska, Bettina Freitag, Sven Meister, Leonard Fehring

**Affiliations:** 1 Faculty of Health, School of Medicine, Witten/Herdecke University, Alfred-Herrhausen-Strasse 50, 58448 Witten, Germany; 2 Health Care Informatics, Faculty of Health, School of Medicine, Witten/Herdecke University, Alfred-Herrhausen-Strasse 50, Witten, 58448 Germany; 3 Department Healthcare, Fraunhofer Institute for Software and Systems Engineering, Dortmund, Germany; 4 Helios Universitätsklinik Wuppertal, Medizinische Klinik 2, Wuppertal, Germany

**Keywords:** Mobile health, mHealth, Digital health, UTAUT2, Technology acceptance

## Abstract

The study aims to (1) investigate current levels of patient acceptance of mHealth in Germany; (2) determine the influencing factors of patients' intention to use, and (3) test the influence of prescription and reimbursement status on patient acceptance. Online survey with 1349 participants, of which 1051 were complete and included for statistical analysis, from a broad cross-section of the German population, addressing both users of mobile health (mHealth) applications and people without prior experience. SEM modeling based on a combination of two theoretical frameworks: the extended Unified Theory of Acceptance and Use of Technology and Health Protective Behavior Theories were used to assess acceptance. Users of mHealth in Germany are mostly patients between the ages of 30 – 50 with mental health or endocrine conditions. General willingness to use mHealth apps / DiGAs (mHealth apps fully reimbursed by social health insurance) is high at 76%, especially if they are governmentally certified, however only 27% of respondents were willing to pay out of pocket. With the exception of a spike in performance expectancy and data security, DiGAs lack a clear differentiation from mHealth apps. Perceived self-efficacy and performance expectancy are significant predictors of willingness to use digital health interventions; with age, attitude, and e-literacy being key demographic predictors. A key takeaway for regulators, providers of mHealth apps/ DiGAs, and other stakeholders involved in mHealth adoption is the importance of addressing negative beliefs early on, targeted communication around effortless usage of mHealth services across age groups and demographics, and focus on highlighting expected benefits of mHealth app/ DiGA usage.

**Supplementary Information:**

The online version contains supplementary material available at 10.1007/s10916-023-01910-x.

## Introduction

As digitalization advances across industries, healthcare systems are increasingly engaging with digital health applications, which also include mobile health (mHealth) [1]. Such technological innovation carries great potential in facilitating patient access to health, reducing cost and improving the effectiveness and efficiency of healthcare systems [2, 3] – especially in the face of globally increasing health costs and shifting demographics [4, 5].

The World Health Organization defines mHealth as the “medical and public health practice supported by mobile devices, such as mobile phones, patient monitoring devices, personal digital assistants, and other wireless devices” [6]. As of March 2022, there were over 104,000 medical applications available globally [7].

To prevent stifling innovation, regulatory bodies have embraced a laissez-faire approach in regard to overseeing mHealth apps [8] in the past. This created an intransparent marketplace for patients, who often lack the specialized knowledge necessary to identify not only which apps are right for them personally but also to judge the adequate price for and the medical benefit of an individual mHealth application [9]. Some offers have remained out of reach for patients due to cost constraints; others misled patients with claims not held up by scientific evidence [9]. To ensure patient safety while at the same time removing barriers to access and promoting mHealth as a valuable treatment addition, the German government passed the DVG (“Digitale-Versorgung-Gesetz”) in October 2019. The law enables physicians to prescribe defined mHealth apps, with statutory health insurance covering the cost for the patient. Such certified apps are then called DiGAs. To attain “DiGA”-status (prescription and reimbursement coverage), mHealth applications must go through a comprehensive certification process and provide scientific evidence for efficacy through clinical trials. Other certification requirements include safety, functional capability, quality, interoperability, data protection and data security [10]. Thus, some of the core hurdles to patient adoption – namely, prohibitive cost, lack of integration with current standard of care and quality concerns [11] – are addressed in the German system, which is unique worldwide. Since 2019, some 90 mHealth applications have applied for certification, with 33 successfully completing the process as of June 2022 (19 preliminary certifications, 12 permanent, and 2 removed after certification). The coverage of these applications extends to various indications, with around half of them focused on providing supportive therapy for mental and behavioral disorders. Core functionalities revolve around patient education, symptom tracking, exercises and training, decision guidance, and behavior recommendations [12].

Despite the increasing number of DiGAs available to patients in Germany, patient adoption has been slow [13]. A recent study examined the hurdles to mHealth adoption from physicians’ and psychotherapists’ point of view, who, as the prescribing authority, play a key role in the implementation process of mHealth under the German system. Of the sample of 1308 prescribers, only 62.1% supported the opportunity to prescribe DiGAs, with main concerns centered around physician’s insufficient information on the app, lack of reimbursement for related medical services such as consultation on how to use the app, insufficient medical evidence as well as legal and technological uncertainties [14]. Nonetheless, patients themselves play a crucial role in adopting mHealth. While a body of past research has focused on identifying hurdles to patient adoption and assessing user acceptance of digital interventions as out-of-pocket add-ons to therapy, the effect of offering fully reimbursed, prescription-based mHealth applications on patient acceptance has not been subject to research yet. The aim of this paper is to (1) investigate current levels of patient acceptance of mHealth applications in Germany; (2) determine the influencing factors of patients' intention to use and (3) test the influence of prescription and reimbursement status on patient acceptance.

## Methods

We used a two-step approach to conduct a web-based based on the UTAUT2 [15] model. First, we conducted a systematic literature review following the PRISMA guidelines [16] to define an adequate model for the acceptance of mHealth in Germany, taking into account the effect of reimbursement and prescription. Second, based on this systematic literature review, we defined the research model. In the next step, we designed a web survey (for the full questionnaire and detailed CHERRIES see Multimedia Appendix 1). Survey questions were based, where possible, on existing, published research; with UTAUT2 [15] questionnaire items based on a German translation [17]. Last, we analyzed the collected data and refined the model using exploratory factor analysis, ANOVA, and SEM modeling using R.

### Systematic literature review

**Fig. 1:**
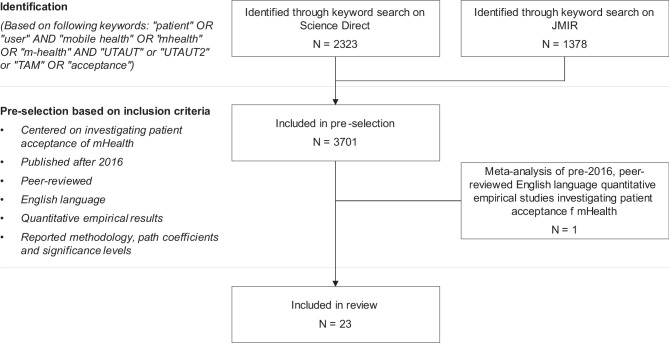
Article selection process

We identified relevant citations using the Science Direct and JMIR database. The search terms were selected based on three factors: 1) focusing on patients, 2) using technology acceptance theories, and 3) centered on mHealth. The initially identified 3701 citations were then screened based on pre-defined inclusion criteria, leading to 23 articles included in the review after screening (article selection process as described in Fig. 1). We included one meta-analysis of articles published prior to 2016, while the remaining 22 citations all comprise quantitative empirical studies published in or after 2016 (for the full review of literature, please see Multimedia Appendix 2).

### Theoretical foundation

A patient’s decision-making process leading up to the usage decision in this context is multi-faceted and complex, as it involves not only technological constraints and considerations but also psychological and cognitive factors particular to individual health behaviors [18]. Previous research has highlighted the importance of applying theories of health behavior to the study of acceptance and usage of mHealth services, as the intention to use mHealth services closely resembles the intention to engage in (protective) health behavior [19]. Underlying conceptual frameworks to assess the use of mHealth applications are thus often an adaptation of well-established technology acceptance models such as the extended UTAUT2 [15], sometimes in combination with Health Protective Behavior Theories such as the health belief model [20] or the protection motivation theory [18, 19, 21–25]. Nonetheless, a best practice to evaluate patient acceptance and expectations for the context of mHealth remains to be established – mHealth being highly context-dependent, be it geographical context or clinical setting. Most published works focus on Asian countries and the US, and while some research has focused on Germany [26], patient acceptance of prescription-based mHealth apps has not been assessed before. The fact that mHealth apps can be prescribed and reimbursed by SHIs significantly shifts the dynamics of patient acceptance or rejection. Factors such as patient-physician relationship, data security and integrity, surveillance anxiety, and self-efficacy gain importance when mHealth apps can be prescribed similarly to pharmaceuticals. To this end, we integrated and built on existing models and adjusted them to fit the specifics of our target geography: a market shaped by being the first worldwide to introduce prescribable mHealth applications (DiGAs).

### The research model

The research model is based on an adaptation of mHealth acceptance models developed by Koivumäki et al. [18] and Rajak and Shaw [27] and combines the Unified Theory of Acceptance and Use of Technology (UTAUT2) [15] and Health Protective Behavior Theories [25]. This way, our research draws from a well-established and tested model with UTAUT2, while also considering irrationalities and specifics in human behavior pertaining to health issues.

Five UTAUT2 model dimensions were included in the research model:

#### Performance expectancy

PE is defined as “the degree to which an individual believes that using a technology will help them to gain a profit in performance” [15]. In this study, performance expectancy refers to an expected improved health outcome. Within existing research, PE is considered the major predictor for patients’ intention to use mHealth services [22, 28–39]. A recent meta-analysis of 67 studies on patient acceptance models within the mHealth space [40] confirms PE as the second-strongest indicator for behavioral intention (β = 0.41); another finds that especially compared with fitness/ wellness applications, PE is a stronger indicator for use within purely medical apps [41].

#### Effort expectancy (EE)

EE is defined as “the degree of simplicity and ease of use of a system” [15]. This construct refers to the perceived ease of use and the learning effort involved in the use of the mHealth app, as well as the ease of access to the technology (reimbursement and prescription). Existing research is less unanimous on the impact of EE on intention to use mHealth apps: some studies have indicated that high EE may negatively influence behavioral intention [18, 22, 29, 31, 33, 35, 37, 42, 43]; with others showing no significant effects [30, 32, 34, 44]. These disparate findings are reflected in the meta-analysis [40], in which ease of use demonstrated a weaker relationship with intention to use (β = 0.21) than e.g., PE.

#### Social influence (SI)

SI is defined as “the degree to which an individual perceives that others (such as peers, authority figures, and family members) believe he or she should use a technology” [15]. In the context of this study, this dimension specifically refers to how far individuals believe physicians, people that are important to the individual, and the general public perceive they should use mHealth apps. SI has been less extensively researched within the mHealth space, nonetheless, several studies have shown that SI has a significant positive influence on behavioral intention [22, 27, 33, 34, 43, 44]. It is interesting to note that a study directly comparing factors influencing behavioral intention between mHealth applications and wellness/ fitness apps finds SI to be the only other determinant next to PE for mHealth app usage [41].

#### Facilitating conditions (FC)

FC expresses “consumers' perceptions of the resources and support available to perform a behavior” [15]. For our purposes, facilitating conditions encompass the integration into users’ existing technical equipment and the integration into the health ecosystem. Research points to a weak positive relationship between FC and intention to use [28, 29, 31–35, 44]; with one study finding FC to be country-agnostic in demonstrating a positive effect on behavioral intention [42]. However, some studies also reported that FC had no significant effect on users’ behavioral intention to use mobile health technologies [22, 43].

#### Hedonic motivation (HM)

This construct is defined as “the user’s pleasure of using a technology” [15]. There is discord regarding the inclusion of this dimension in the context of health behavior, as this usually is not connected to pleasurable experiences, with the primary outcome not geared toward entertainment, but positive health outcomes. Nonetheless, most research indicates that HM is one of the important factors in predicting the intention to use technologies across disciplines [15, 45]. Some recent research included this dimension in the context of mHealth and technology acceptance and found it to have a weak positive impact on intention to use [46], especially in light the Covid-19 pandemic. Another study found HM to overlap with performance expectancy in the context of mHealth apps, as users commented on the app’s performance and effort expectancy when asked about the likeability and enjoyability of a mHealth app [45]. In our study, besides the standard definition as “the user’s pleasure of using a system” [15], we have also included a slightly modified definition: “a user’s positive feelings associated with the use of the app” to attenuate the concept of “pleasure”.

We excluded habit, as patients require a sufficient amount of time to formulate a habitual behavior towards mHealth service [22]; in addition, the model aims to assess the behavioral intention of individuals both with and without use experience of mHealth technology. We did not include price value, as it implies first a monetary cost attached to the use and second the users’ awareness of this cost as well as comparability between mHealth providers. In Germany, there is a lack of cost associated for users of DiGAs and no “average/ reasonable” price for mHealth apps due to the infant nature of the technology.

In the UTAUT2 model, age, gender, and experience are posited to moderate the impact of the key constructs on usage intention. We added self-assessed electronic literacy to mediate effects stemming from low experience with using mobile technologies, on which mHealth interventions are based.

The decision-making process behind using mHealth services can be compared to engaging in health protective behaviors, thus, we have included two dimensions from the Protection Motivation Theory (PMT) into the research model: response cost or perceived barriers to taking a health-related action and self-efficacy. There is evidence that consumers associate some risks with the use of mHealth technologies; chief among them privacy concerns and data security [11, 18, 47, 48]. To capture the effect of perceived barriers – especially those related to prescription by physicians and reimbursement by SHIs – as broadly as possible, our research model synthesizes and includes various barriers discussed by previous studies: information risk, data quality concerns, personal impediments, trust/ relationship interference, technology risk and change resistance [18, 22, 27, 29, 41].

Based on the literature review (for the full literature review, please see Multimedia Appendix 2), the following hypotheses were developed:

- H 1: Performance expectancy will influence behavioral intention positively
- H 2: Effort expectancy will influence behavioral intention positively
- H 3: Social influence will influence behavioral intention positively
- H 4: Facilitating conditions will influence behavioral intention positively
- H 5: Hedonic motivation will influence behavioral intention positively
- H 6: Self-efficacy will influence behavioral intention positively
- H 7a: Data security and quality concerns will influence behavioral intention negatively
- H 7b: Attitude will influence behavioral intention positively
- H 8: Age will influence behavioral intention negatively
- H 9: Experience will influence behavioral intention positively
- H 10: Self-assessed electronic literacy will influence behavioral intention positively

### Sample composition, survey design and questionnaire

The convenience sample includes 1051 respondents (out of the total 1349 respondents, having removed incomplete or incorrectly answered responses as well as IP address duplicates) from a broad cross-section of the German population, with both users of DiGAs and mHealth apps and people without prior usage experience. Written informed consent was provided by all participants. Responses indicating “I do not know” were excluded from statistical analysis.

The survey was approved and compliance with GDPR guidelines (DSGVO in German) was verified by the Ethics Committee of Witten/ Herdecke University (Nr. S-288/2021). Prior to administering the survey, a pre-test with a sample of 15 participants was conducted to test the questionnaire for clarity, comprehensiveness, and technical functionality.

The survey took place between February 15 and May 31, 2022. Recruitment channels included social networks such as Facebook and LinkedIn, online platforms for research, providers of digital health applications, and prescribers. The target was to recruit a broad sample of the German population.

### Empirical analyses

Data was analyzed with the software R (version 4.1.3) with the add-on packages “psych” and “dplyr” for ANOVA and post hoc testing, and “lavaan” for SEM modeling. Estimations were made using diagonally weighted least squares (DWLS) based on a polychoric correlation matrix [49]. Exploratory factor analysis (EFA) was used to confirm and identify optimal factors among the dimensions added to the established UTAUT2 model constructs (self-efficacy and perceived barriers to use). EFA identified 3 optimal factors: SEff (self-efficacy, or the “extent to which one believes one is able to perform a behavior”, related to both health and technological self-efficacy) was confirmed as a stand-alone factor, while two individual categories for perceived barriers were found: data security and quality concerns, and attitude (which is comprised of trust, technology resistance, and other personal impediments).

## Results

**Fig. 2:**
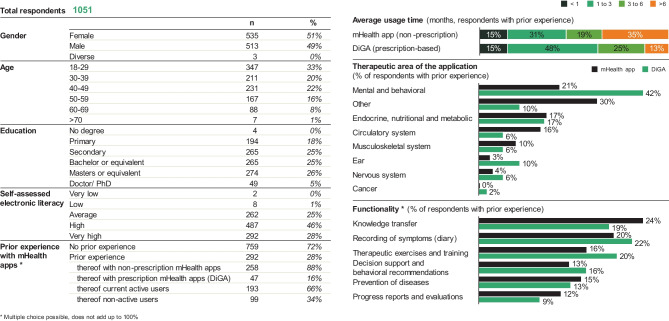
Demographics

Overall, we recruited a broad sample of 1051 German respondents with a mean age of 39 years (SD 13.5) and diverse educational backgrounds. Most of the sample indicated above-average self-assessed electronic literacy: 28% indicated very high, 46% high, and 25% average (for more detail, see Fig. 2).

### Users of mHealth in Germany are mostly patients between the ages of 30 – 50 with mental health or endocrine conditions

**Fig. 3:**
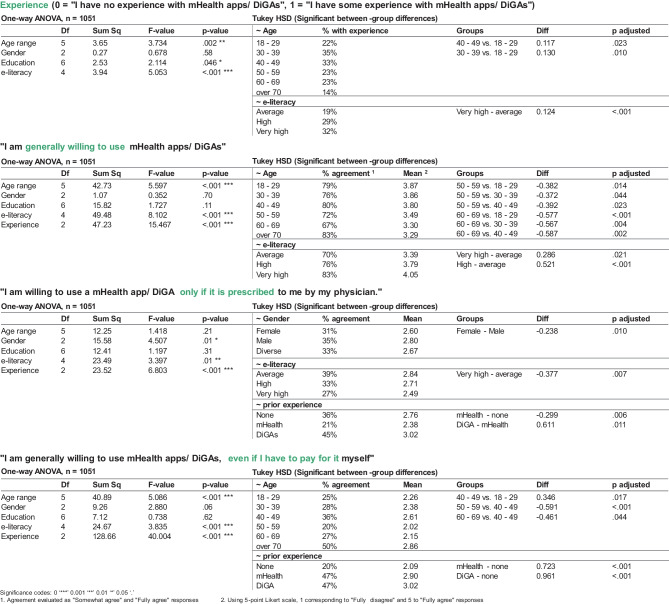
Selected ANOVA and post-hoc analyses results

Overall, 292 (27%) respondents had prior experience with mHealth applications/ DiGAs, out of which 47 (16%) either were current users or had used prescription-based DiGAs at some point. Of those with prior experience, the majority (232, 79.5%) were aged below 50; 29% of this age group had experience using mHealth applications/ DiGAs when compared to 23% of those aged 50 and above. It is interesting to note that testing for variance between groups (ANOVA and Tukey HSD, see Fig. 3) within the under 50 age group, respondents aged 30 – 50 were significantly likelier to have prior experience with mHealth/ DiGAs (35% of 30 – 30 age group and 33% of 40 – 49 age group) than their younger counterparts aged 18 – 29 (22%, *P* = 0.01 resp. 0.023). Respondents with higher self-assessed e-literacy were significantly likelier to have prior mHealth/ DiGA experience: respondents who reported “very high” e-literacy were 60% more likely to have prior experience than those with “average” self-reported scores (32% vs. 20%, *P* = 0.009). There were no significant differences in mHealth/ DiGA experience based on gender or education.

When looking into therapeutic areas, most mHealth/ DiGA users had experience with apps for either mental and behavioral (21% of non-prescription mHealth app users and 42% of DiGA users) or endocrine and nutritional disorders (17% for both). It is of note that DiGAs show a clear spike in the mental and behavioral disorder space when compared to non-prescription mHealth apps, which can be ascribed to the number of DiGAs in this therapeutic area (19/31 available DiGAs as of June 2022) and the care gap within this space in Germany (on average, patients wait 20 weeks from their initial request to start a psychotherapeutic treatment [50]).

Average usage times for non-prescription mHealth apps were slightly higher than for DiGAs: only 13% of DiGA-users indicated they used the app 6 months or longer compared to 35% of non-prescription mHealth app users. The most frequently mentioned reasons to stop using mHealth apps/ DiGAs included “I do not need it anymore” (33%), “I do not find it helpful to use” (31%), and “I do not have time to use it” (24%). When comparing responses for DiGA users to non-prescription mHealth app users, it is worth noting that “I do not find it helpful to use” was only mentioned by 14% of DiGA-experienced users (vs. 34% mHealth app users), while “the usage is too complicated” was not mentioned at all (vs. 8% mHealth app users).

### General willingness to use mHealth apps / DiGAs is high, especially if they are governmentally certified, however only 27% were willing to pay out of pocket

76% of respondents would generally be willing to use mHealth applications/ DiGAs, with younger age (*P* < 0.001, standardized β = -0.083) and higher self-assessed e-literacy (*P* < 0.001, standardized β = 0.14) being predictors of intention to use (see Fig. 3). ANOVA and Tukey HSD show there are no statistically significant differences between the age groups of 18 – 49: as a whole, this age range is significantly more likely to use mHealth compared to older generations (see Fig. 3).

**Fig. 4:**
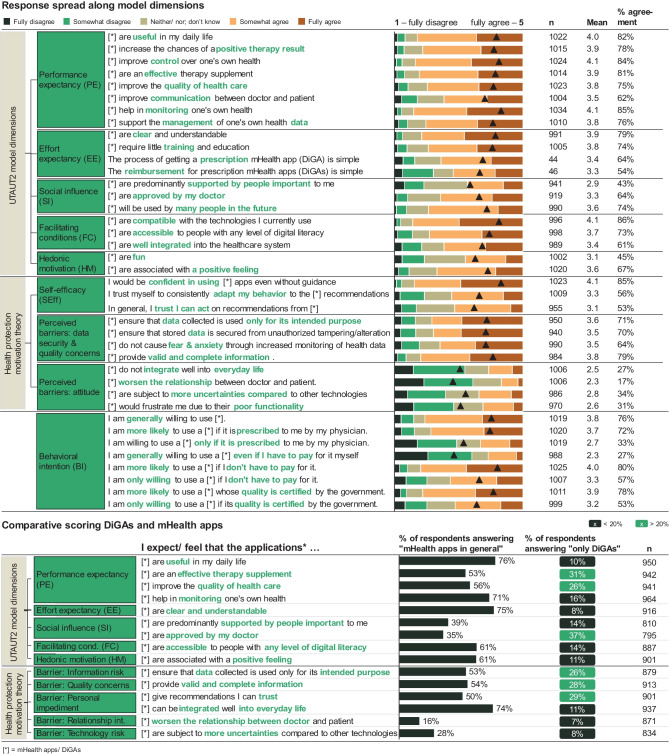
Response spread along model dimensions and comparative scoring DiGAs and mHealth apps

While 53% of respondents would use mHealth apps/ DiGAs only if their quality were certified by the government, prescription by physicians is overall only somewhat important: 33% state they would use only if prescribed (see Fig. 4). However, there are significant differences when it comes to prescription needs between genders (*P* = 0.01), e-literacy (*P* = 0.01), and prior experience (*P* < 0.001). Prescription is more important to men than women (35% of men would use only if prescribed vs 31% of women, *P* = 0.01), while lower e-literacy implies a higher need for prescription by a physician (39% “average” self-reported e-literacy vs 27% “very high”, *P* = 0.007). Users experienced with DiGAs report the highest need for prescription by physicians: 45% state they would use mHealth only if prescribed.

When looking into the willingness to pay, only 27% of respondents would be willing to use a mHealth app/ DiGA if they had to pay out-of-pocket. There are statistically significant differences between age groups (*P* < 0.001) and prior experience (*P* < 0.001): willingness to pay peaks in the age group 40 – 49 (36% would be willing to pay) and decreases for both older and younger populations (20% for ages 50 – 59, *P* < 0.001; and 26% for ages 18 – 29, *P* = 0.017).

### DiGAs lack a clear differentiation from mHealth apps

When asked to compare mHealth applications to prescription-based DiGAs, respondents only see differentiation criteria in three main areas: medical performance, physician acceptance, and data quality and security (see Fig. 4). 76% of respondents believe mHealth apps in general to be useful in their daily life. 31% state that only DiGAs are an effective therapy supplement (vs. 53% mHealth apps in general). Similarly, around ¼ of respondents believe only DiGAs provide complete and valid information and give recommendations they could trust (compared to ½ mHealth apps). The sole dimension in which “only DiGAs” outperform mHealth apps is physician approval, with 37% of respondents stating “only DiGAs are approved by my doctor”.

### Perceived self-efficacy and performance expectancy are significant predictors of willingness to use digital health interventions

**Fig. 5:**
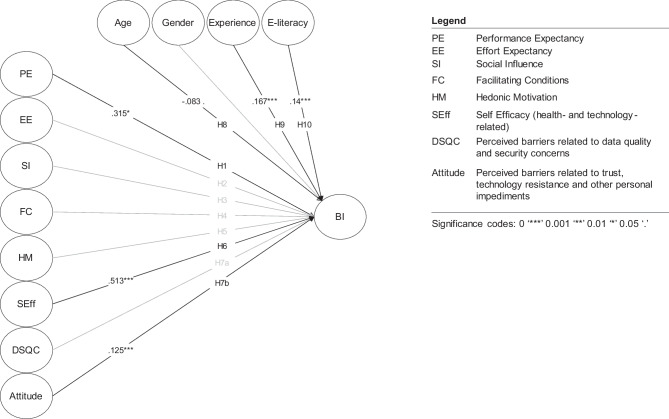
SEM model with standardized path coefficients & conceptual framework of the study

Modeling intention to use as a function of the UTAUT2-model constructs, self-efficacy (SEff), and perceived barriers (see Fig. 5), we see that only performance expectancy, self-efficacy, and attitude have a significant effect on intention to use mHealth/ DiGAs (standardized path coefficients = 0.513 for SEff, 0.315 for PE, and 0.125 for attitude). Of the control variables, age has a small negative effect on the intention to use mHealth/ DiGAs, whereas prior experience and higher e-literacy both exhibit a moderate positive effect (see Fig. 5), confirming H8, H9, and H10. We also confirm hypotheses H1, H6, and H7b, discarding H2 through H5 and H7a, meaning that facilitating conditions, social influence, hedonic motivation, and data security and quality concerns do not affect respondents’ intention to use mHealth/ DiGAs (see Fig. 5).

**Fig. 6:**
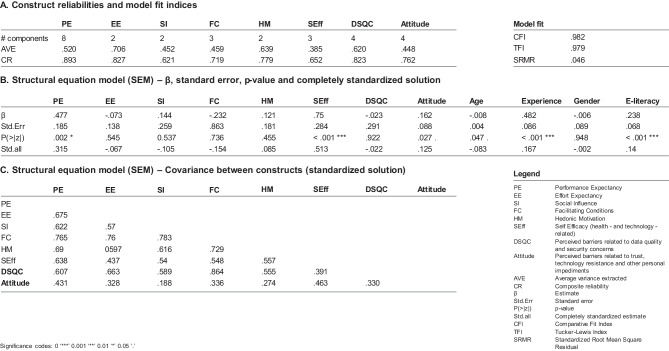
Construct reliabilities, model fit indices and SEM model output

Taking into account theoretical justifications and fit indices (RMSEA = 0.033, SRMR = 0.046, CFI = 0.982, TLI = 0.979), the suggested research model is significant [51–53]. We used convergent validity and discriminant validity to assess construct reliability (see Fig. 6). All constructs meet the acceptable standards for construct reliability for exploratory research [54].

## Discussion

### Users, usage, and user experience

Overall, the respondent demographic is a broad sample of the German population and confirms recent findings on mHealth/ DiGA usage in Germany, with around 1/3 of respondents having previous experience with mHealth apps and 5% with DiGAs [55]. The observed link between lower age, higher electronic literacy and mHealth/ DiGA usage has also been proven to be significant by past research [36, 56].

Looking into shorter usage times for DiGAs as compared to mHealth, it is important to note that the former have been available for a shorter period of time. Thus, it would be misleading to draw any conclusions as to user experience or adherence based on usage time alone. When comparing the reasons to stop using mHealth apps to DiGAs, it becomes clear that experiences differ depending on whether the app in question is a prescription-based DiGA or a non-prescribed mHealth app. The main reasons to stop mHealth app usage revolve around lack of need, helpfulness, and time constraints, with app quality and ease of use playing a minor role. Contrary to that, DiGA users mainly stop using their app due to lack of need or time and data security concerns, with app functionality or ease of use playing no role at all. This can be explained by the different motivations for using mHealth apps/ DiGAs: prescription-based DiGAs are used for a very specific need, sometimes for limited timeframes, for a diagnosed medical condition exclusively. The data input for DiGAs is often highly sensitive and personal, such as specific daily symptoms, increasing user concerns regarding data privacy and security. Compared to that, many mHealth apps are rather used to fulfill lifestyle needs and are often less targeted towards specific conditions (e.g. nutrition apps, cycle-tracking). As previous research shows[41], when the medical need for mHealth apps is less pronounced, ease of use becomes more important.

It would be interesting to further investigate methods to increase patient adherence: a recent study [57] found healthcare professionals to have the greatest potential to promote patient adherence to digital therapeutics, however, the correlation between patient adherence and app design remains relatively unexplored.

### Willingness to use, prescription, certification, and willingness to pay

The overall high willingness to use mHealth apps/ DiGAs (76%) is a positive sign for app providers and proponents of digitalization in the healthcare space. The highest adoption rates can be expected for the ages 30 – 49, with no significant differences between genders. High self-assessed electronic literacy additionally supports willingness to use, stressing the need for patient education. It is interesting to note that physician prescription plays a minor role only, with 67% of respondents willing to use mHealth apps/ DiGAs that are not prescribed. This is particularly interesting considering the rather skeptical stance of German physicians regarding mHealth apps/ DiGAs – in a recent survey among German healthcare professionals, only 30.3% (393/1299) planned to prescribe DiGA [14]; another found 31% believe digitization endangers the trust in the doctor-patient relationship [55]. As app reimbursement is possible either through physician prescription or through approval by the health insurer, app providers could focus on strengthening relationships with insurers and building patient awareness to improve adoption rates.

On the other side, quality certification by a government or other entity is more important to patients (53% would only be willing to use mHealth apps/ DiGAs when their quality is certified by the government, see Fig. 4). This might be due to the fact that multiple applications for the same indication are available, and patients lack the specialized knowledge to identify the appropriate app with proven medical benefit. The need for more transparency and quality checks within mHealth has been highlighted by numerous studies [8, 47, 48], especially in light of some cases of misleading statements by app providers and the lack of proven medical benefits of apps [9]. This transparency can be provided by different stakeholders: e.g., in Germany, the Central Institute for the Provision of Health Care by Statutory Health Insurance (German: “Zentralinstitut für die kassenärztliche Versorgung in der Bundesrepublik Deutschland”) provided a KV app radar (a portal which synthesizes and aggregates app store reviews and where both users of mHealth apps/ DiGAs and physicians can additionally review and comment on applications). Additional actions could include introducing an open-source directory of app evidence or standardized facts labels for health apps.

Despite the willingness to pay out of pocket for mHealth apps/ DiGAs being relatively low (only one in four participants would be willing to pay, on average), it is interesting to note that it increases for users experienced with mHealth apps and then again for users experienced with DiGAs. This is particularly important in light of the current discussions on pricing dynamics for DiGAs in Germany: currently, app developers set prices for DiGAs through negotiations with the GKV-SV (“Gesetzlicher Krankenversicherungs Spitzenverband”, German statutory health insurance association), with price limits sets based on comparators within one indication group (prices being considered too high if they exceed 80% of comparators). Although there are few published studies on actual mHealth app/ DiGA usage and adherence, the GKV-SV reported that between September 2020 – 2021, only 80% of prescribed apps were activated [58]. This has led to increased calls from payors to adapt the current reimbursement model to incentivize adherence – be it through the inclusion of value-based elements, co-pay for patients, or other alternatives. Existing research points toward an inverse association between co-pay and medication adherence for pharmaceutical therapies [59]; however, the effects of co-pay on adherence to mHealth apps yet remain to be explored. A recent study found a positive relationship between higher up-front costs and health club attendance [60], mainly due to higher perceived loss for non-attendance.

### Differentiation between DiGAs and mHealth apps

As the first country worldwide to introduce prescription-based digital health interventions covered by SHI, Germany is pioneering digitalization in healthcare. However, based on our study findings, there is room for improvement regarding the communication and public awareness building about mHealth and DiGAs. Except for a spike in performance expectancy and data security and quality, respondents seem to see little difference between prescription-based DiGAs and non-prescribed mHealth apps. This however becomes significant when looking into factors predicting mHealth/ DiGA usage: performance expectancy is the second-strongest driver of intention to use. DiGA providers should thus focus on even further increasing patient awareness about health benefits and expected medical outcomes.

### Predictors of mHealth usage

According to our findings, only performance expectancy, self-efficacy and attitude have a significant effect on the intention to use. Previous research has consistently identified performance expectancy as one of the core predictors of intention to use mHealth apps [22, 28–36]. A recent meta-analysis of 67 studies on patient acceptance models within the mHealth space [40] confirms PE as the second-strongest indicator for behavioral intention (β = 0.41); another finds that especially compared with fitness/ wellness applications, PE is a stronger indicator for use within purely medical apps [41]. This highlights the need for mHealth app developers and regulators to focus on clearly communicating the expected benefits of mHealth app/ DiGA usage first and foremost.

Increasingly, more recent studies have focused on self-efficacy as a significant predictor of intention to use [18, 31]. Improving electronic literacy and health education, which have been identified as determinants of self-efficacy [18] are thus key to boosting mHealth/ DiGA adoption rates. This could be done by traditional methods (training, demonstrations, free trials), but also through targeted marketing communication showcasing effortless usage of mHealth services across age groups and demographics. Considering the situation in the German market, ensuring health care professionals’ remuneration for training patients is key. Under the current system, despite being the key access point [14] to prescription-based DiGAs, remuneration for initial DiGA prescription amounts to only 2€ per patient (GOP 01,470) and 7.21 € for progress monitoring (GOP 01,471/ 01,472 and “Pauschale” 86,700).

The last significant predictor of intention to use identified in our model is attitude. This latent construct is based on trust, technology resistance, and other personal impediments (such as belief about the ability to integrate usage into daily routines) and was confirmed through EFA. Despite the rather loose definition of this dimension, previous research indicates a strong relationship between pre-conceived notions regarding mHealth and the intention to use [18, 30, 40]. A recent study among 2011 German citizens [55] found that almost 1 in 4 respondents believes technology creates more problems than it solves, pointing toward a high overall technology skepticism among the German population. It is important to note here that as previous research points out, technology skepticism tends to be country-specific [61], meaning the results obtained might not translate to different geographies. Nonetheless, a key takeaway for regulators, providers of mHealth apps/ DiGAs, and other stakeholders involved in mHealth adoption is the importance of addressing negative beliefs early on.

Considering the factors identified as not significant on intention to use mHealth technologies/ DiGAs, perhaps the only puzzling aspect is the non-significance of data security and quality concerns. Germany tends to be seen as one of the countries with the strongest attitudes toward data privacy and protection [62], translating to particularly restrictive GDPR. The non-significance of this dimension could be explained by two factors: first, mHealth technology remains relatively new, meaning most respondents in our sample are not experienced in using it. This could translate to a lack of importance placed on data security as users are not aware of which data would be collected and thus do not consider it worthy of protection. Second, users with existing conditions might place more weight on the perceived benefit of digital interventions and be less concerned with data privacy, as mHealth solutions cover a previously unmet need. Additionally, a cross-sectional survey of 1003 adults in Germany revealed a high willingness to share health-related data for research purposes [63] during the Covid-19 pandemic, pointing toward changing attitudes toward data privacy.

Looking into the other non-significant UTAUT2 factors (effort expectancy, social influence, facilitating conditions, and hedonic motivation), previous research is ambiguous as to their impact on intention to use. Effort expectancy is in some instances considered significant for older populations only [43] and often found to not affect the intention to use [30, 32, 34, 44]. This could be explained by the increased penetration of mobile technologies across all age groups and demographics, further strengthened by the Covid-19 pandemic.

Social influence has been less extensively researched within the mHealth space. Some papers indicate a weak positive relationship [22, 33, 34, 43, 44] between the degree to which an individual perceives others believe they should use a technology and the intention to use it. However, this could be explained by most of these studies being conducted in Asian geographies, where the impact of social influence is considered greater due to higher power distance and a less individualistic culture [64].

Facilitating conditions gain importance with increased technology complexity, which leads to a higher need for support infrastructure. In line with previous findings [22, 43], mobile apps have become increasingly integrated into daily routines and smartphone usage has reached sufficient penetration to negate the importance of such support systems; with users placing value on self-efficacy (i.e., the extent to which they believe themselves to be able to perform a behavior that leads to a valued outcome) instead on the ability to simply use mHealth technologies. This is further supported by the positive ratings respondents give for the ease to obtain and receiving reimbursement for a prescription-based DiGA (means of 3.4 and 3.3 out of 5, see Fig. 4).

As to hedonic motivation, there is discord regarding the inclusion of this dimension in the context of health behavior, as this usually is not connected to pleasurable experiences. The primary outcome is not geared toward entertainment, but positive health outcomes. Despite some existing research indicating a weak positive impact on intention to use [46], this ambivalence could help explain the lack of a significant relationship between hedonic motivation and intention to use mHealth apps/ DiGAs in our model.

### Limitations & further research

Although the present study reveals important findings, it has several limitations, the first of which is selection bias, as common for web-based research. This may have resulted in a bias towards populations with higher electronic literacy and exacerbated self-selection bias (skew towards respondents with higher interest in mHealth topics). Second, social influence and hedonic motivation were found not to have a significant effect on the intention to use mHealth, which should be re-examined through further research. Additionally, it would be interesting to examine the effect of price value on intention to use, as users’ awareness of mHealth cost increases with rising mHealth adoption rates.

As opposed to the original UTAUT study, which was a longitudinal study, this research only measures the respondents’ perceptions and intention to use at a single time point. Further research examining perceptions and intention to use over time would be required, especially given the large impact Covid-19 had on patient attitudes toward digital therapies, data sharing, and mHealth.

Finally, although data was collected from a broad population sample in Germany, we cannot claim validity in other countries.

### Conclusion

In conclusion, acceptance of mHealth interventions in Germany is high, with age, high electronic literacy, and prior experience being predictors of the intention to use. Performance expectancy, self-efficacy of the app, and attitude are major levers in improving mHealth adoption, as they have a significant effect on the intention to use. A key takeaway for regulators, providers of mHealth apps/ DiGAs, and other stakeholders involved in mHealth adoption is the importance of addressing negative beliefs early on, targeted communication around effortless usage of mHealth services across age groups and demographics, and focusing on highlighting expected benefits of mHealth app/ DiGA usage.

### Supplementary Information

Below is the link to the electronic supplementary material.

Supplementary file1 (PDF 236 KB)

Supplementary file2 (PDF 170 KB)

## Data Availability

The data that support the findings of this study are available from the corresponding author upon reasonable request.

## Abbreviations

BfArM: German Federal Institute for Drugs and Medical Devices (“Bundesamt für Arzneimittel und Medizinprodukte “)
DiGA: German state-certified digital health application (“Digitale Gesundheitsanwendung”)
mHealth: Mobile health
GOP: Gebührenordnungsposition, “fee order item “, denoting a specific reimbursable service under the remuneration system for SHI-accredited physicians and psychotherapists in Germany
GKV-SV: Gesetzlicher Krankenversicherungs Spitzenverband, German statutory health insurance association
