# Supplementary material for: Patient Acceptance of Prescribed and Fully Reimbursed mHealth Apps in Germany: An UTAUT2-based Online Survey Study
Source: J Med Syst. 2023 Jan 27;47(1):14. doi: 10.1007/s10916-023-01910-x (PMC9880914; doi:10.1007/s10916-023-01910-x)
Supplement: Supplementary file 1 — Supplementary file1 (PDF 236 KB) [file 10916_2023_1910_MOESM1_ESM.pdf]

Multimedia Appendix 1: Survey Design and Questionnaire

Checklist for Reporting Results of Internet E-Surveys (CHERRIES)

Design

The survey consists of 4 sections: (1) demographics (age, gender, education, and self-assessed electronic literacy), (2) previous experience with mHealth apps and prescription-based, fully reimbursed mHealth apps (“Digitale Gesundheitsanwendungen” or “DiGAs”, based on the (“Digitale-Verordnung-Gesetz” from October 2019), (3) attitude towards mHealth apps/ DiGAs, (4) intention to use either or both. The target respondents include a broad cross-section of German population, with both users of DiGAs and mHealth apps and people without prior usage experience to capture as broad a sentiment as possible. The convenience sample includes 1051 respondents (having removed incomplete or incorrectly answered responses as well as IP address duplicates). We included both single and multiple-choice questions for the first two sections and used 5-point Likert scales (“Fully disagree”, “Somewhat disagree”, “neither/ nor”, “Somewhat agree”, “Fully agree”) for the latter two sections. Both “Somewhat agree” and “Fully agree” were evaluated as consent. An “I don’t know” field was provided for all questions using the Likert scale.

IRB approval and informed consent process

The survey was approved and compliance with GDPR guidelines (DSGVO in German) was verified by the Ethics Committee of the university of Witten/ Herdecke (Nr. S-288/2021). Participants were informed via a short introductory text about length of the survey, data security and storage policies, the researchers involved and their individual affiliations, as well as the purpose of the research. Written informed consent was provided by all participants. Data was anonymized and stored solely on the server of the University Witten/ Herdecke. No personal information was collected that could be tied back to individual respondents. Participation was voluntary.

Development and pre-testing

The survey was developed based on extensive review of existing literature in the context of user acceptance of digital health/ mHealth. To identify relevant papers, research from the year 2000 onwards including the keywords “acceptance“, „technology acceptance”, “UTAUT”, “UTAUT2”, “TAM”

in combination with “mHealth” “digital health”, “health app(lication)”, “digital therapeutics” or DTx” was included. Survey questions were based, where possible, on existing, published research; with UTAUT2 questionnaire items based on a German translation [1]. Prior to administering the survey, a pre-test with a sample of 15 participants was conducted to test the questionnaire for clarity, comprehensiveness, and technical functionality. Based on the pre-test, the wording of several questions was adjusted, especially regarding the clear differentiation between mHealth apps and prescription based DiGAs. Additionally, a visual aid was provided to help participants distinguish between both. The survey took place between February 15, 2022, and May 31, 2022.

Recruitment process and description of the sample having access to the questionnaire

The survey was conducted in an open mode, meaning any person with an access link could participate in the survey. Initial contact was made via the Internet, with the following main recruitment channels:

- social networks such as LinkedIn or Facebook, using both the researcher’s own network and independent groups (groups included, but were not limited to: residents of major German cities and regions; specific age categories such as i.e., students, senior citizens; indications such as i.e., mental health issues or insomnia; interests groups such as sports, digitalization or health; professional groups such as i.e., teachers),
- online platforms for research and academic communications, such as. i.e., thesius.com
- mailing lists (both publicly available mailing lists for research purposes and personal contacts),
- providers of digital health applications
- prescribers

Announcements used were nuanced and varied in length to fit the audience and channel employed:

- On **LinkedIn**, the survey was shared with the following message: “Was bringen DiGA wirklich? Patienten-Umfrage zur Akzeptanz von digitalen Gesundheitsanwendungen. Jetzt teilnehmen: [surveylink]” or “ "Patientenakzeptanz und Nutzung digitaler Gesundheitsanwendungen" – In einem mehrstufigen wissenschaftlichen Untersuchungsprozess der Technologieakzeptanzforschung widmet sich Marie Uncovska, u.a. Promovendin an der Universität Witten/Herdecke, dieser Frage. Gerne beteiligen unter: [surveylink]“.

- **Facebook** messages were less formal and included variations of: “App auf Rezept - Helfer oder Humbug? Teile Deine Meinung hier: [surveylink]” or “App als Therapieregänzung bei [indication]? Für ein Forschungsvorhaben sucht die Universität Witten/Herdecke noch Teilnehmer für eine Befragung zu Gesundheitsapps (ca. 5 Minuten). Aktuell gibt es vermehrt Apps, die als digitaler Helfer bei der Therapie unterstützen sollen. Was haltet ihr davon? Habt ihr Erfahrung in dem Bereich gemacht? Anbei ist der Link zur Teilnahme: [surveylink]“.
- **Research platforms and mailing lists** included a more detailed description of the research: “App auf Rezept - Helfer oder Humbug? Für ein Forschungsvorhaben sucht die Universität Witten/ Herdecke noch Teilnehmer für eine kurze, 5-minütige Online-Umfrage. Ziel der Forschung ist es, das Meinungsbild zu Gesundheitsapps innerhalb der breiten deutschen Bevölkerung zu erfassen. Die Umfrage richtet sich an alle deutschen Bundesbürger, unabhängig von ihrer Vorerfahrung mit digitalen Gesundheitsanwendungen. Klicken Sie hier, um an der Umfrage teilzunehmen: [surveylink]“. An information box regarding data privacy and data security was also included in these messages.
- **Providers of digital health applications and prescribers** were contacted via phone or e-mail and then disseminated the survey with their own announcements through their channels.

Survey administration

The survey was administered solely through the web. The above-described recruitment process is not without limitations: 1) the non-representative nature of the Internet population, 2) the self-selection of participants (volunteer effect) and 3) a potential bias toward participants with an existing interest in mHealth applications. Nonetheless, we sought to counter these biases by targeting as diverse a population as possible and selecting a wide variety of recruitment channels. This is also reflected in the composition of the sample, with a roughly equal spread between the genders, ages, and education levels. Further bias was avoided by a lack of incentives provided as well as the voluntary nature of the survey. As previously described, the questionnaire consisted of 4 sections, displayed accordingly on 4 pages. The total amount of questions was 15, with section 1 containing four single-choice questions, section 2 seven single- or multiple-choice questions, section 3 three questions to be answered via Likert scale with a total of 47 sub-categories and section 4 one question to be answered via Likert-scale with 7 sub-categories. Adaptive questioning was implemented for several questions based on respondents’ prior experience with mHealth apps to reduce the number and

complexity of the questions. Respondents were able to review and change their responses through a “back” button at the bottom of each screen. The selection of a response option was enforced (mandatory responses for all questions displayed to the respondent), with non-response options (“I do not know”) provided.

Response rates

A total of 1349 respondents clicked on the survey link, with 1121 of those fully completing the questionnaire, leading to a completion rate of 83%.

Preventing multiple entries from the same individual

An IP check was used to identify duplicate entries from the same user. A total of 42 duplicate IP addresses was identified and removed from the sample, reducing the total number of questionnaires to 1079.

Analysis

Only complete questionnaires were used for analysis. To ensure the quality of responses, a test question was included in the questionnaire. Only responses with a correctly answered test question were included in the analysis, leading to an additional elimination of 28 responses. The total number of responses thus included in the analysis was 1051. No statistical correction was used to adjust the sample.

Original German language survey version

<u>Begrüßungstext</u>

Vielen Dank, dass Sie sich etwa 5 Minuten für die Beantwortung dieser Umfrage nehmen.
Die Studie untersucht die Einstellung und Erfahrungen der deutschen Bevölkerung zu Gesundheitsapps allgemein sowie zu staatlich zugelassene digitalen Gesundheitsanwendungen (DiGAs). **Gesundheitsapps sind Anwendungen zur Prävention sowie Behandlung von Krankheiten**, für die Arztsuche und den direkten Kontakt zum Arzt oder auch zur Gesundheitsförderung durch Stressabbau oder eine gesunde Ernährung.

Bei DiGAs handelt es sich um spezielle, staatlich zugelassenen Gesundheitsapps, die von Ärzten und Psychotherapeuten als „digitale Helfer" verordnet werden und die die Erkennung, Überwachung, Behandlung oder Linderung von Krankheiten mittels einer digitalen Plattform (Smartphone-App, Web-basierte Plattformen) unterstützen. **Die Kosten** für die DiGA sowie für ggfs. im Rahmen ihrer Anwendung erforderliche ärztliche Leistungen werden von **der gesetzlichen Krankenversicherung übernommen.**

Die Studie richtet sich an deutsche Bundesbürger und hat zum Ziel, das Stimmungsbild zur Nutzung von Gesundheitsapps und staatlich zugelassenen DiGAs zu erfassen, sowie Hürden aus Patientensicht zu identifizieren. Darauf basierend können Empfehlungen zur Optimierung dieser generiert werden. Die Umfrage richtet sich sowohl an Nutzer von Gesundheitsapps und staatlich zugelassenen DiGAs als auch an Menschen ohne vorherige Erfahrung mit diesen, da ein möglichst breites Stimmungsbild erfasst werden soll.

Sie bleiben bei Ihrer Teilnahme anonym. Die hier erhobenen Daten werden rein für wissenschaftliche Zwecke genutzt und sind nicht zu Ihrer Person nachverfolgbar. Aufgrund der anonymen Datenerhebung ist eine nachträgliche Löschung Ihrer Daten nicht möglich.

Checkbox: Ich stimme den <u>Datenschutzbestimmungen</u> zu und nehme an der Studie freiwillig teil.

Section 1: Demographics

1. Bitte geben Sie ihr Alter in Jahren an. *(Answered as numeric input)*

2. Bitte geben Sie Ihr Geschlecht an. *(Answered as single-choice question)*
    a. Weiblich
    b. Männlich
    c. Divers

3. Was ist Ihr höchster Schul- oder Hochschulabschluss? *(Answered as single-choice question)*
    a. Kein Abschluss
    b. Hauptschulabschluss
    c. Mittlere Reife
    d. Abitur oder gleichwertiger Abschluss
    e. Bachelor-Abschluss
    f. Master-Abschluss / Diplom
    g. Doktor/ PhD

4. Wie schätzen Sie ihre allgemeine digitale Kompetenz ein? *(Answered as single-choice question)*
    a. Sehr hoch
    b. Hoch
    c. Durchschnittlich
    d. Niedrig
    e. Sehr niedrig

Section 2: Previous experience with mHealth apps/ DiGAs

5. Welche der folgenden Aussagen trifft auf Ihre Erfahrung mit Gesundheitsapps/ DiGAs am ehesten zu? *(Answered as multiple-choice question)*
    a. Ich verwende aktuell eine DiGA, die mir vom Arzt verschrieben wurde
    b. Ich verwende aktuell mindestens eine Gesundheitsapp, die keiner Verschreibung vom Arzt bedarf
    c. Ich habe in der Vergangenheit eine mir vom Arzt verschriebene DiGA verwendet, aktuell nutze ich diese nicht mehr
    d. Ich habe in der Vergangenheit eine nicht verschreibungspflichtige Gesundheitsapp verwendet, aktuell nutze ich diese nicht mehr
    e. Ich habe keine Erfahrung mit Gesundheitsapps/ DiGAs

6. [If answered (5) with a, c]: Mit welcher der folgenden DiGAs haben Sie Erfahrung? *(Answered as single-choice question; drop-down menu)*
    a. CANKADO PRO-React Onco
    b. companion patella powered by medi
    c. deprexis
    d. elevida
    e. EYSTA
    f. HelloBetter
    g. Invirto

h. Kalmeda
i. M-sense
j. Mawendo
k. Mika
l. Mindable
m. NichtraucherHelden
n. Novego
o. Oviva Direkt
p. Rehappy
q. Selfapy
r. Somnio
s. Velibra
t. Vivira
u. Vorvida
v. Zanadio

7. [If answered (5) with a, b, c, d]: Welchem Bereich lässt sich die Gesundheitsapp/ DiGA, mit der Sie vertraut sind, am ehesten zuordnen? *(Answered as single-choice question)*
    a. Psychische und Verhaltensstörungen
    b. Krankheiten des Nervensystems
    c. Krebs
    d. Endokrine, Ernährungs- und Stoffwechselkrankheiten
    e. Krankheiten des Kreislaufsystems
    f. Krankheiten des Muskel-Skelett-Systems und des Bindegewebes
    g. Krankheiten des Ohrs
    h. Sonstige [freies Textfeld]

8. [If answered (5) with a, b, c, d]: Welche Funktionalitäten weist die Gesundheitsapp/ DiGA, mit der Sie vertraut sind, auf? *(Answered as multiple-choice question)*
    a. Prävention von Krankheiten
    b. Wissensvermittlung
    c. Erfassen von Symptomen („Tagebuch“)
    d. Therapeutische Übungen und Trainings
    e. Entscheidungshilfen und Verhaltensempfehlungen
    f. Fortschrittsberichte und Auswertungen
9. [If answered (5) with a, b, c, d]: Über welchen Zeitraum verwenden Sie/ haben Sie die Gesundheitsapp/ DiGA verwendet? *(Answered as single-choice question)*
    a. 0 – 1 Monat
    b. 1 – 3 Monate
    c. 3 - 6 Monate
    d. Länger als 6 Monate

10. [If answered (5) with c, d]: Aus welchem Grund haben Sie aufgehört, die Gesundheitsapp/ DiGA zu benutzen? *(Answered as multiple-choice question)*

a. Ich brauche sie nicht mehr
b. Ich empfinde die Nutzung nicht als hilfreich
c. Die App ist nicht relevant für mich
d. Die Qualität der Inhalte der App ist schlecht
e. Die Nutzung ist zu kompliziert
f. Ich habe keine Zeit für die Verwendung
g. Ich habe Zweifel an der Datensicherheit
h. Die Nutzung führt bei mir zu Stress
i. Ich habe eine Verschlechterung der Beziehung zu meinem behandelnden Arzt beobachtet
j. Die Funktionsweise der App ist schlecht
k. Sonstige [freies Textfeld]

11. [Test question] Testfrage: Bitte wählen Sie „stimme überhaupt nicht zu“ aus.*(Answered as single-choice question through 5-point Likert scale; “Fully disagree”, “Somewhat disagree”, “Neither/ nor”, “Somewhat agree”, “Fully agree”)*

Section 3: Attitude towards mHealth Apps/ DiGAs

12. Ich erwarte/ empfinde, dass die Nutzung von Gesundheitsapps/ DiGAs... *(Answered as single-choice question through 5-point Likert scale; “Fully disagree”, “Somewhat disagree”, “Neither/ nor”, “Somewhat agree”, “Fully agree”; non-response option provided as “I do not know”)*

**ID**	**Question**	**Source**
PE1	... im Alltag nützlich ist	Venkatesh, 2012
PE2	... die Chancen auf ein positives Therapieergebnis erhöht	New item
PE3	... die Kontrolle über die eigene Gesundheit verbessert	New item
PE4	... eine effektive Therapieergänzungen darstellt	van der Vaart, 2016
PE5	... die Qualität der Gesundheitsvorsorge verbessert	van der Vaart, 2016
PE6	... die Kommunikation zwischen Arzt und Patient verbessert	van der Vaart, 2016
PE7	... bei der Überwachung der eigenen Gesundheit hilft	Lin et al, 2011
PE8	... bei der Pflege der eigenen Gesundheitsdaten unterstützt	Deng, 2013
EE1	... einfach und verständlich ist	Venkatesh, 2012
EE2	... wenig Übung und Aufklärung verlangt	van der Vaart, 2016
SI1	... von mir wichtigen Personen überwiegend befürwortet wird	Venkatesh, 2012
SI2	... von meinem Arzt befürwortet wird	van der Vaart, 2016
HM1	... Spaß macht	Venkatesh, 2012
HM2	... mit einem positiven Gefühl verbunden ist	New item
FC1	... kompatibel mit den Technologien, die ich aktuell verwende, ist	Venkatesh, 2012
FC2	... durch vorhandenes Aufklärungsmaterial für Menschen mit jeder digitalen Kompetenz zugänglich ist	Venkatesh, 2012
FC3	... gut in das Gesundheitssystem integriert ist	van der Vaart, 2016
B-IR1	... gewährleisten, dass die erhobenen Daten ausschließlich für den vorgesehenen Zweck verwendet werden.	Deng, 2013
B-IR2	... sicherstellen, dass die gespeicherten Daten vor unbefugter Manipulation/Veränderung gesichert sind.	Deng, 2013
B-QC1	... gültige und vollständige Informationen zur Verfügung stellen.	Deng, 2013
B-PI1	... keine Ängste und Sorgen durch verstärkte Überwachung von Gesundheitsdaten verursachen.	New item
B-TR1	... im Vergleich zu anderen Technologien mit mehr Unsicherheiten behaftet sind.	Palacholla, 2019

B-PI2	... sich nicht gut in den Alltag integrieren lassen.	Deng, 2013
B-RI1	... das Verhältnis zwischen Arzt und Patient verschlechtern.	Palacholla, 2019
B-TR1	... mich aufgrund ihrer schlechten Funktionalität frustrieren würden.	Palacholla, 2019

13. Inwieweit stimmen Sie folgenden Aussagen zu? *(Answered as single-choice question through 5-point Likert scale; "Fully disagree", "Somewhat disagree", "Neither/ nor", "Somewhat agree", "Fully agree"; non-response option provided as "I do not know")*

EE3	Der Prozess eine verschreibungspflichtige Gesundheitsapp (DiGA) zu erhalten ist für mich unkompliziert	New item
EE4	Der Kostenübernahmeprozess für staatlich zugelassene Gesundheitsapps (DiGAs) ist einfach	New item
SI3	Gesundheitsapps/ DiGAs werden künftig von vielen Menschen genutzt werden	van der Vaart, 2016
SE - Tech	Ich würde mir zutrauen, Gesundheitsapps/ DiGAs richtig zu nutzen, auch wenn niemand da wäre, der mir zeigt, wie man sie nutzt	Deng, 2013
SE - Health	Ich würde mir zutrauen, mein Verhalten konsequent an die Empfehlungen der Gesundheitsapp/ DiGA anzupassen	Bandura,
B-CR1	Ich möchte nicht, dass Gesundheitsapps/ DiGAs die Art und Weise ändern, wie ich mit gesundheitlichen Fragen umgehe	Hoque & Sorwar, 2017
SE	Im Allgemeinen vertraue ich darauf, Empfehlungen von Gesundheitsapps/ DiGAs umsetzen zu können	Klaver et al., 2021

14. Ich erwarte/ empfinde, dass die Anwendung… *(Answered as single choice question, with following answer options: "mHealth apps in general", "Only DiGAs", "Neither/ Nor", "I don't know")*

PE1	... im Alltag nützlich ist	Venkatesh, 2012
PE2	... eine effektive Therapieergänzungen darstellt	van der Vaart, 2016
PE3	... die Qualität der Gesundheitsvorsorge verbessert	van der Vaart, 2016
PE4	... bei der Überwachung der eigenen Gesundheit hilft	Lin et al, 2011
EE1	... einfach und verständlich ist	Venkatesh, 2012
SI1	... von mir wichtigen Personen überwiegend befürwortet wird	Venkatesh, 2012
SI2	... von meinem Arzt befürwortet wird	van der Vaart, 2016
HM1	... beim Benutzen mit einem positiven Gefühl verbunden ist	New item
FC1	... durch vorhandenes Aufklärungsmaterial für Menschen mit jeder digitalen Kompetenz zugänglich ist	Venkatesh, 2012
B-RI1	… Empfehlungen gibt, denen ich vertrauen würde	Klaver et al., 2021
B-IR1	... gewährleistet, dass die erhobenen Daten ausschließlich für den vorgesehenen Zweck verwendet werden	Deng, 2013
B-QC1	... gültige und vollständige Informationen zur Verfügung stellt	Deng, 2013
B-PI1	... sich gut in den Alltag integrieren lässt	Deng, 2013
B-RI1	... das Verhältnis zwischen Arzt und Patient verschlechtert	Palacholla, 2019
B-TR1	... im Vergleich zu anderen Technologien mit mehr Unsicherheiten behaftet ist	Palacholla, 2019

Section 4: Intention to use mHealth apps/ DiGAs

15. Inwieweit stimmen Sie folgenden Aussagen zu? *(Answered as single-choice question through 5-point Likert scale; "Fully disagree", "Somewhat disagree", "Neither/ nor", "Somewhat agree", "Fully agree"; non-response option provided as "I do not know")*

BI1	Ich wäre allgemein dazu bereit, Gesundheitsapps zu verwenden.	Venkatesh, 2012
BI2	Es ist wahrscheinlicher, dass ich eine Gesundheitsapp verwende, wenn sie mir vom Arzt verschrieben wird.	New item
BI3	Ich wäre nur dann dazu bereit, eine Gesundheitsapp zu verwenden, wenn sie mir von meinem Arzt verschrieben wird.	New item
BI4	Ich wäre allgemein dazu bereit, eine Gesundheitsapp auch dann zu verwenden, wenn ich selbst dafür bezahlen muss	New item
BI5	Es ist wahrscheinlicher, dass ich eine Gesundheitsapp verwende, wenn ich nichts dafür bezahlen muss.	New item
BI6	Ich wäre nur dann dazu bereit, eine Gesundheitsapp zu verwenden, wenn ich nicht dafür bezahlen muss.	New item
BI7	Es ist wahrscheinlicher, dass ich eine Gesundheitsapp verwende, deren Qualität staatlich zertifiziert ist.	New item
BI8	Ich wäre nur dann dazu bereit, eine Gesundheitsapp zu verwenden, wenn deren Qualität staatlich zertifiziert ist.	New item

Translated English language survey version

Welcome text

Thank you for taking about 5 minutes to respond to this survey.

The study investigates the German population's acceptance of and experiences with both non-prescribed mHealth apps and prescription-based digital mHealth applications (DiGAs). mHealth apps are applications for the prevention as well as treatment of diseases, used for e.g., booking and conducting (digital) physician appointments, disseminating medical knowledge or tracking health-relevant information.

DiGAs are a sub-class of mHealth apps that are government-approved and prescribed by physicians and psychotherapists as "digital helpers" that support the detection, monitoring, treatment or alleviation of diseases using a digital platform (smartphone app, web-based platforms). The costs for the DiGA as well as for any medical services required in the context of its use are covered by the statutory health insurance.

The study is aimed at all German citizens independently of previous experience with either app type and aims to capture patient acceptance of and intention to use mHealth apps, as well as to identify hurdles to adoption. Based on this, recommendations for optimizing them can be generated.

Participants will remain anonymous. The data collected here will be used purely for scientific purposes and will not be traceable to individuals. Due to the anonymous data collection, a subsequent deletion of individual data sets is not possible.

Checkbox: I agree to the privacy policy and participate in the study voluntarily.

Section 1: Demographics

1. Please enter your age in years. *(Answered as numeric input)*
2. Please indicate your gender. *(Answered as single-choice question)*
    a. Female
    b. Male
    c. Diverse
3. Please indicate your education level. *(Answered as single-choice question)*

a. No degree
b. Primary
c. Secondary (9th year)
d. Secondary (12/13th year)
e. Bachelor or equivalent
f. Masters or equivalent
g. Doctor/ PhD

4. How do you rate their overall digital literacy? *(Answered as single-choice question)*
    a. Very high
    b. High
    c. Average
    d. Low
    e. Very low

Section 2: Previous experience with mHealth apps/ DiGAs

5. Which of the following statements is most applicable to your experience with mHealth apps/ DiGAs? *(Answered as multiple-choice question)*
    a. I am using at least one mHealth application (DiGA) that was prescribed to me by my physician
    b. I am using at least one mHealth application that required no prescription
    c. I have used at least one mHealth application (DiGA) that was prescribed to me by my physician in the past
    d. I have used at least one mHealth application that required no prescription in the past
    e. I have no experience with mHealth apps/ DiGAs

6. [If answered (5) with a, c]: Which of the following DiGAs do you have experience with? *(Answered as single-choice question; drop-down menu)*
    a. CANKADO PRO-React Onco
    b. companion patella powered by medi
    c. deprexis
    d. elevida
    e. EYSTA
    f. HelloBetter
    g. Invirto
    h. Kalmeda
    i. M-sense
    j. Mawendo
    k. Mika
    l. Mindable
    m. NichtraucherHelden
    n. Novego
    o. Oviva Direkt
    p. Rehappy
    q. Selfapy
    r. Somnio

s. Velibra
t. Vivira
u. Vorvida
v. Zanadio

7. [If answered (5) with a, b, c, d]: To which area does the health app/ DiGA you are familiar with most closely align? *(Answered as single-choice question)*
    a. Mental and behavioral
    b. Nervous system
    c. Cancer
    d. Endocrine, nutritional and metabolic
    e. Circulatory system
    f. Musculoskeletal system
    g. Ear
    h. Other [free text field]

8. [If answered (5) with a, b, c, d]: Which functionalities does the health app/ DiGA you are familiar with have? *(Answered as multiple-choice question)*
    a. Prevention of diseases
    b. Knowledge transfer
    c. Recording of symptoms (diary)
    d. Therapeutic exercises and training
    e. Decision support and behavioral recommendations
    f. Progress reports and evaluations

9. [If answered (5) with a, b, c, d]: Over what period have you been using/ have you used the mHealth app/ DiGA? *(Answered as single-choice question)*
    a. 0 – 1 months
    b. 1 – 3 months
    c. 3 - 6 months
    d. 6 months or longer

10. [If answered (5) with c, d]: Why did you stop using the mHealth app/ DiGA? *(Answered as multiple-choice question)*
    a. I do not need it anymore
    b. I do not find it helpful to use
    c. The app is not relevant for me
    d. The quality of the app's content is poor
    e. The usage is too complicated
    f. I do not have time to use it
    g. I have doubts about data security
    h. The use causes me stress
    i. It had a negative effect on the relationship with my attending physician
    j. The functioning of the app is poor
    k. Other [free text field]

11. [Test question] Test question: Please select "fully disagree". *(Answered as single-choice question through 5-point Likert scale; "Fully disagree", "Somewhat disagree", "Neither/ nor", "Somewhat agree", "Fully agree")*

Section 3: Attitude towards mHealth Apps/ DiGAs

12. I expect/feel that the use of mHealth apps/ DiGAs... *(Answered as single-choice question through 5-point Likert scale; "Fully disagree", "Somewhat disagree", "Neither/ nor", "Somewhat agree", "Fully agree"; non-response option provided as "I do not know")*

ID	Question	Source
PE1	[*] are useful in my daily life	Venkatesh, 2012
PE2	[*] increase the chances of a positive therapy result	New item
PE3	[*] improve control over one's own health	New item
PE4	[*] are an effective therapy supplement	van der Vaart, 2016
PE5	[*] improve the quality of health care	van der Vaart, 2016
PE6	[*] improve communication between doctor and patient	van der Vaart, 2016
PE7	[*] help in monitoring one's own health	Lin et al, 2011
PE8	[*] support the management of one's own health data	Deng, 2013
EE1	[*] are clear and understandable	Venkatesh, 2012
EE2	[*] require little training and education	van der Vaart, 2016
SI1	[*] are predominantly supported by people important to me	Venkatesh, 2012
SI2	[*] are approved by my doctor	van der Vaart, 2016
HM1	[*] are fun	Venkatesh, 2012
HM2	[*] are associated with a positive feeling	New item
FC1	[*] are compatible with the technologies I currently use	Venkatesh, 2012
FC2	[*] are accessible to people with any level of digital literacy	Venkatesh, 2012
FC3	[*] are well integrated into the healthcare system	van der Vaart, 2016
B-IR1	[*] ensure that data collected is used only for its intended purpose	Deng, 2013
B-IR2	[*] ensure that stored data is secured from unauthorized tampering/alteration	Deng, 2013
B-QC1	[*] provide valid and complete information	Deng, 2013
B-PI1	[*] do not cause fear and anxiety through increased monitoring of health data	New item
B-TR1	[*] are subject to more uncertainties compared to other technologies	Palacholla, 2019
B-PI2	[*] do not integrate well into everyday life	Deng, 2013
B-RI	[*] worsen the relationship between doctor and patient	Palacholla, 2019
B-TR2	[*] would frustrate me due to their poor functionality	Palacholla, 2019

13. To what extent do you agree with the following statements? *(Answered as single-choice question through 5-point Likert scale; "Fully disagree", "Somewhat disagree", "Neither/ nor", "Somewhat agree", "Fully agree"; non-response option provided as "I do not know")*

EE3	The process of getting a prescription mHealth app (DiGA) is simple.	New item
EE4	The reimbursement process for prescription mHealth apps (DiGAs) is simple.	New item
SI3	[*] will be used by many people in the future	van der Vaart, 2016

SE - Tech	I would be confident in using [*] apps even without guidance	Deng, 2013
SE - Health	I trust myself to consistently adapt my behavior to the [*] recommendations	Bandura,
B-CR1	I would not want [*] to change the way I deal with health issues	Hoque & Sorwar, 2017
SE	In general, I trust I can act on recommendations from [*]	Klaver et al., 2021

14. I expect/feel that the use of mHealth apps/ DiGAs... *(Answered as single choice question, with following answer options: "mHealth apps in general", "Only DiGAs", "Neither/ Nor", "I don't know")*

PE1	[*] are useful in my daily life	Venkatesh, 2012
PE2	[*] are an effective therapy supplement	van der Vaart, 2016
PE3	[*] improve the quality of health care	van der Vaart, 2016
PE4	[*] help in monitoring one's own health	Lin et al, 2011
EE1	[*] are clear and understandable	Venkatesh, 2012
SI1	[*] are predominantly supported by people important to me	Venkatesh, 2012
SI2	[*] are approved by my doctor	van der Vaart, 2016
HM1	[*] are associated with a positive feeling	New item
FC1	[*] are accessible to people with any level of digital literacy	Venkatesh, 2012
B-RI1	[*] give recommendations I can trust	Klaver et al., 2021
B-IR1	[*] ensure that data collected is used only for its intended purpose	Deng, 2013
B-QC1	[*] provide valid and complete information	Deng, 2013
B-PI1	[*] can be integrated well into everyday life	Deng, 2013
B-RI1	[*] worsen the relationship between doctor and patient	Palacholla, 2019
B-TR1	[*] are subject to more uncertainties compared to other technologies	Palacholla, 2019

Section 4: Intention to use mHealth apps/ DiGAs

15. To what extent do you agree with the following statements? *(Answered as single-choice question through 5-point Likert scale; "Fully disagree", "Somewhat disagree", "Neither/ nor", "Somewhat agree", "Fully agree"; non-response option provided as "I do not know")*

BI1	I am generally willing to use [*]	Venkatesh, 2012
BI2	I am more likely to use a [*] if it is prescribed to me by my doctor	New item
BI3	I am willing to use a [*] only if it is prescribed to me by my doctor	New item
BI4	I am generally willing to use a [*] even if I have to pay for it myself	New item
BI5	I am more likely to use a [*] if I don't have to pay for it	New item
BI6	I am only willing to use a [*] if I don't have to pay for it	New item
BI7	I am more likely to use a [*] whose quality is certified by the government	New item
BI8	I am only willing to use a [*] if its quality is certified by the government	New item

References:

Bandura, A. (2004). Health promotion by social cognitive means. Health Education and Behavior, 31(2), 143–164. https://doi.org/10.1177/1090198104263660

Deng, Z. (2013). Understanding public users' adoption of mobile health service. International Journal of Mobile Communications, 11(4), 351-373.

Harborth, D., & Pape, S. (2018). German translation of the unified theory of acceptance and use of technology 2 (UTAUT2) questionnaire. Available at SSRN 3147708.

Hoque, R., & Sorwar, G. (2017). Understanding factors influencing the adoption of mHealth by the elderly: An extension of the UTAUT model. International journal of medical informatics, 101, 75-84.

Klaver, N. S., van de Klundert, J., & Askari, M. (2021). Relationship between perceived risks of using mHealth applications and the intention to use them among older adults in the Netherlands: cross-sectional study. JMIR mHealth and uHealth, 9(8), e26845

Koivumäki, T., Pekkarinen, S., Lappi, M., Väisänen, J., Juntunen, J., & Pikkarainen, M. (2017). Consumer adoption of future MyData-based preventive eHealth services: an acceptance model and survey study. Journal of medical Internet research, 19(12), e429.

Palacholla, R. S., Fischer, N., Coleman, A., Agboola, S., Kirley, K., Felsted, J., ... & Jethwani, K. (2019). Provider-and patient-related barriers to and facilitators of digital health technology adoption for hypertension management: scoping review. JMIR cardio, 3(1), e11951

van der Vaart, R., Atema, V., & Evers, A. W. (2016). Guided online self-management interventions in primary care: a survey on use, facilitators, and barriers. BMC family practice, 17(1), 1-9.

Venkatesh, V., Thong, J. Y., & Xu, X. (2012). Consumer acceptance and use of information technology: extending the unified theory of acceptance and use of technology. MIS quarterly, 157-178.

Abbreviations/ Abkürzungsverzeichnis

PE: Performance Expectancy - Leistungserwartung
EE: Effort Expectancy - Aufwandserwartung
SI: Social Influence – Sozialer Einfluss
HM: Hedonic Motivation – Hedonistische Motivation
FC: Facilitating Conditions – Erleichternde Bedingungen
BI: Behavioral Intention – Verhaltensabsicht
IR: Information risk – Informationsrisiko
QC: Quality concerns - Qualitätsbedenken
PI: Personal impediments – Persönliche Beeinträchtigungen
RI: Relationship interference
TR: Technology risk
CR: Change resistance
