# Supplementary material for: Patient Acceptance of Prescribed and Fully Reimbursed mHealth Apps in Germany: An UTAUT2-based Online Survey Study
Source: J Med Syst. 2023 Jan 27;47(1):14. doi: 10.1007/s10916-023-01910-x (PMC9880914; doi:10.1007/s10916-023-01910-x)
Supplement: Supplementary file 2 — Supplementary file2 (PDF 170 KB) [file 10916_2023_1910_MOESM2_ESM.pdf]

Multimedia Appendix 2: Review of existing literature on mHealth acceptance

Authors	DOI/ access link	Methods	Geography	Sample size	Model	Findings	Effect on BI…					
							PE	EE	FC	SI	HM	SE
Alam, M. Z., Hoque, M. R., Hu, W., & Barua, Z. 2020	10.1016/j.ijinfomgt.2019.04.016	Survey among Gen Y	Bangladesh	296	UTAUT plus price and perceived reliablity	Results confirmed that performance expectancy, social influence, facilitating conditions and perceived reliability positively influence the behavioral intention to adopt mHealth services. However, effort expectancy and price value did not have a significance influence on the behavioral intention. Moreover, gender has a significant moderating effect on mHealth services adoption in certain cases	yes	no	yes	yes		
Boontarig, 2016	10.1109/ICSEC.2016.7859897	Survey among social media users	Thailand	212	UTAUT model and Big Five personality traits as moderators	Findings indicate that facilitating conditions and perceived value had a significant effect on behavioral intention to use m- Health information through social media. Conscientiousness acts as moderator for facilitating conditions and perceived value	yes	yes	yes			
Breil et al., 2019	10.3233/SHTI190839	Survey among hypertension patients in Germany and Austria	Germany & Austria	145	UTAUT plus self-efficacy, openness to experience and perceived health threat were analyzed as predictors.	145 participants (mean age 52.51 years, SD 14.33; 60% female) completed the survey. Acceptance was moderate on average (M = 3.26, SD = 1.07, min 1 to max 5). In a multiple hierarchical regression, performance expectancy and effort expectancy were confirmed as significant predictors of acceptance (step 1, R2 = .57, p < .001), while self-efficacy could not be confirmed (step 2, p = .87). In addition, perceived health threat (β = .12, p < .05) and openness to experience (β = .22, p < .001) had a significant influence on acceptance of mHealth apps for hypertension (step 3, overall model with R2 = .62). Age showed a negative association with the intention to use (β = .22, p = .005) while no influence of gender could be found (p = .06).	yes	yes				no
Cimperman, Makovec Brenčič, & Trkman, 2016	10.1016/j.ijmedinf.2016.03.002	Survey among citizens aged 50 and above	Slovenia	400	UTAUT plus doctor's opinion, computer anxiety, and perceived security as contextual predictors	Performance expectancy, effort expectancy, facilitating conditions, and perceived security were confirmed having a direct impact on behavioral intention to use home telehealth services.	yes	yes	yes			
Dhiman, N., Arora, N., Dogra, N., & Gupta, A. 2020.	10.1108/JIBR-05-2018-0158	Survey among fitness app users	India	324	UTAUT2 plus perceived value, self-efficacy and personal innovativeness	Significant predictors of smartphone fitness app adoption intention include effort expectancy, social influence, perceived value, habit and personal innovativeness. Further, this study confirms significant relationship between personal innovativeness and habit, self-efficacy and effort expectancy and effort expectancy and performance expectation. This study reveals that personal innovativeness is the strongest predictor of	no	yes	no	yes	no	no

Authors	DOI/ access link	Methods	Geography	Sample size	Model	Findings	Effect on BI…					
							PE	EE	FC	SI	HM	SE
						behavioural intention. Contrary to the expectations, factors like performance expectancy, facilitating conditions and hedonic motivation did not influence .behavioural intention						
Duarte, P., & Pinho, J. C. 2019.	10.1016/j.jbusres.2019.05.022	Survey among mHealth users	Portugal	120	UTAUT2	The findings are that performance expectancy (PE) plus habit (HT), and performance expectancy (PE) plus hedonic motivation (HM) are actually necessary conditions for mHealth adoption.					yes	
Dwivedi, Shareef, Simintiras, Lal, & Weerakkody, 2016	10.1016/j.giq.2015.06.003	Survey among diabetic patients	Bangladesh, US, Canada	1158	UTAUT2 plus waiting time and self-concept	Hedonic motivation was not significant for the US and Canadian users, self-concept was significant for Bangladeshi users. Effort expectancy and facilitating conditions are the two major contributors regardless of the country.		yes	yes		no	
Dzimiera, 2017	https://repositorio.ucp.pt/handle/10400.14/22772	Broad population survey	Germany	295	UTAUT plus self-efficacy, privacy and security risk, surveillance anxiety, and physical risk.	Effort expectancy, privacy and security risk, and physical risk have no significant effect on the behavioral intention to use mHealth. Performance expectancy is the major predictor. Self- efficacy was found to be a good predictor of effort expectancy (R2 = 0.639).	yes	no				yes
Hoque & Sorwar, 2017	10.1016/j.ijmedinf.2017.02.002	Broad population survey	Bangladesh	274	UTAUT plus technology anxiety and resistance to change	The study determined that performance expectancy, effort expectancy, social influence, technology anxiety, and resistance to change have a significant impact on the users' behavioral intention to adopt mHealth services. Conversely, facilitating condition revealed having no significant relation with behavioral intention to use mHealth.	yes	yes	no	yes		
Idrish, Rifat, Iqbal, & Nisha, 2017	10.4018/IJTHI.2017040101	Broad population survey	Bangladesh	908	UTAUT plus personal innovativeness, perceived self-efficacy, and perceived financial cost	Findings suggest self-efficacy, facilitating conditions, effort expectancy and performance expectancy to influence users' behavioral intention to adopt mobile health services, with age and gender acting as moderators.	yes	yes	yes			yes
Jewer, 2018	10.1016/j.ijmedinf.2018.01.008	Survey among hospital patients	Canada	118	UTAUT	The results show significant effects in performance expectancy and facilitating conditions on behavioral intention to use an emergency department wait-times website, while the effort expectancy impact was not found significant.	yes	no	yes			
Kenny & Connolly, 2017	ISBN: 9780991556700	Survey	Ireland, US	450	Performance expectancy and social	The model explained 65.5% of the variance in adoption intention for the whole sample. The mHealth self-efficacy and health status did not present a significant effect on the intention to adopt mHealth.						no

Authors	DOI/ access link	Methods	Geography	Sample size	Model	Findings	Effect on BI…					
							PE	EE	FC	SI	HM	SE
					influence from UTAUT plus mHealth self-efficacy, healthcare need, and health status							
Koivumäki et al., 2017	10.2196/jmir.7821	Survey among faculty and staff of university of Oulu	Finland	855	UTAUT2 plus health belief model	Effort expectancy (beta=.191, P<.001), self-efficacy (beta=.449, P<.001), threat appraisals (beta=.416, P<.001), and perceived barriers (beta=−.212, P=.009) are significant predictors of intention to use mHealth technologies.		yes				yes
Macedo, 2017	10.1016/j.chb.2017.06.013	Survey among adults aged 55 and older	Portugal	278	UTAUT2	Results confirm that most UTAUT2 predictors, some of them directly (behavioral intention, habit) and others indirectly (performance expectancy, effort expectancy, social influence, facilitating conditions, hedonic motivation) influence intention to use mHealth.		yes	yes	yes	yes	
Moon & Hwang, 2016	10.1007/978-3-662-47895-0_38	Survey among college students	Korea	126	UTAUT plus personal innovativeness and perceived enjoyment	Findings suggest that social influence positively affects user intention to use, and that performance expectancy is positively correlated with the intention to use. Perceived enjoyment positively affects the potential intention to use the services. Personal innovativeness, effort expectancy, and facilitating conditions did show a significant effect on intention to use.	yes	no	yes	yes	yes	
Nisha, Iqbal, & Rifat, 2019	10.4018/JGIM.2019010102	Survey among urban smartphone users	Bangladesh	927	UTAUT plus system quality (system reliability, system	Facilitating conditions was found to be the strongest direct determinant in influencing behavioral intention of mHealth services adoption. Additionally, effort expectancy, facilitating conditions, performance expectancy, and trust were also statistically found to be predictors of the behavioral intention to use mHealth		yes	yes			
Nunes, Limpo & Castro, 2019	10.3389/fpsyg.2019.02791	Survey study among smarthone users that do not user mHealth apps	Portugal	574	UTAUT	Results showed that the intention to use mobile health applications was determined by performance expectancy moderated by age and smartphone experience, and that the role of the other determinants depended on age and gender (e.g., more intention to use in older men if less effort, and in younger men if better facilitating conditions). These findings show that user characteristics are relevant moderators and should be considered when targeting specific populations to use mobile health applications.	yes					
Quaosar, Hoque, & Bao, 2018	10.1089/tmj.2017.0111	Broad population survey	Bangladesh	245	UTAUT plus perceived credibility	Findings indicate that performance expectancy, effort expectancy, social influence, and perceived credibility significantly influence the elderly's intention to use mHealth services. Facilitating conditions has no significant influence.	yes	yes	no	yes		

Authors	DOI/ access link	Methods	Geography	Sample size	Model	Findings	Effect on BI…					
							PE	EE	FC	SI	HM	SE
Rajak & Shaw, 2021	10.1016/j.techsoc.2021.101800	Survey among mHealth service users in India	India	289	TAM plus SI, technology anxiety, trust, perceived risk, perceived physical condition, resistance to change	The adoption of mHealth was found to have had an enormous impact on social influence, behavioural intention and trust.				yes		
Ravangard, Kazemi, Zaker Abbasali,Sharifian, & Monem, 2017	10.19082/3862	Survey among users of laboratory e-sevices	Iran	170	Hedonic motivation, price value, and habit of the UTAUT2 plus usability, and ability to use the technology	The findings suggest that price value, hedonic motivation, habit, and usability have a significant influence on the intention to use mHealth portals.					yes	
Salgado, Tavares & Oliveira, 2020	10.2196/17588	Survey among students and chronically diseased patients	Portugal	322	UTAUT2 + personal empowerment	The drivers of behavior intention with statistical significance were performance expectancy (β=.29, P<.001), habit (β=.39, P<.001), and personal empowerment (β=.18, P=.01). The precursors of use behavior were habit (β= .47, P<.001) and personal empowerment (β=.17, P=.01). Behavior intention to recommend was significantly influenced by behavior intention (β=.58, P<.001) and personal empowerment (β=.26, P<.001). The model explained 66% of the total variance in behavior intention, 54% of the variance in use behavior, and 70% of the variance in behavior intention to recommend.	yes					
Schomakers, E. M., Lidynia, C., & Ziefle, M. 2019	10.1007/978-3-319-94619-1_6	Broad population survey	Germany	165	UTAUT2 and selected barriers (privacy concerns)	Use intention, performance expectancy (PE), effort expectancy (EE), social influence (SI), facilitating conditions (FC), as well as privacy concerns for a fitness app (lifestyle context) and a diabetes app (medical context). Structural equation modeling is used to assess the relevance of influences on adoption intention in these contexts. Results show that acceptance factors indeed differ strongly between lifestyle and medical contexts. For the latter, only PE and SI determine intention to use, although privacy concerns are higher than in the lifestyle context.				yes		
Tao et al., 2020	10.1016/j.chb.2019.09.023	Meta-analysis			TAM	Attitude was the strongest predictor of behavioral intention (β = 0.64), followed by perceived usefulness (β = 0.41), self-efficacy (β = 0.24), perceived ease of use (β = 0.21), subjective norm (β = 0.19), trust (β = 0.17), and	yes	yes	no	yes		

Authors	DOI/ access link	Methods	Geography	Sample size	Model	Findings	Effect on BI…					
							PE	EE	FC	SI	HM	SE
						perceived behavioral control (β = 0.14). Perceived usefulness could be predicted by subjective norm (β = 0.23) and trust (β = 0.43), while perceived ease of use could be predicted by self-efficacy (β = 0.51). Specifically, all the synthesized relationships from the two types of coefficients were positive and statistically significant, except that the synthesized correlation of facilitating conditions- behavioral intention is significant, while its synthesized path coefficient is non- significant.						
